# Crystal and mol­ecular structures of datiscetin and its monohydrate isolated from *Datisca cannabina* L.

**DOI:** 10.1107/S2056989026004056

**Published:** 2026-04-29

**Authors:** Dilnoza Sh. Akhmedova, Kambaral K. Turgunov, Sabir Z. Nishanbaev

**Affiliations:** a S. Yunusov Institute of the Chemistry of Plant Substances Academy of Sciences of Uzbekistan Mirzo Ulugbek Str 77 Tashkent 100170 Uzbekistan; Institute of Chemistry, Chinese Academy of Sciences

**Keywords:** *Datisca cannabina* L., datiscetin, flavonoids, crystal structure, X-ray diffraction

## Abstract

The crystal structures of the flavonoid datiscetin [3,5,7-trihy­droxy-2-(2-hy­droxy­phen­yl)chromen-4-one], C_15_H_10_O_6_, and its monohydrate, C_15_H_10_O_6_·H_2_O, were determined by single-crystal X-ray diffraction analysis. The flavonoid was isolated from the ethyl acetate fraction of *Datisca cannabina* L. collected in Tashkent region, Uzbekistan. In both structures, the chromenone ring system and the phenyl ring form nearly the same dihedral angles (38.3 and 38.7° for the anhydrate and monohydrate, respectively). In addition, in both structures, the mol­ecules stack through π–π inter­actions along the shortest translation axis of the unit cell [3.7601 (1) and 3.9274 (5) Å, respectively], resulting in the formation of needle-shaped single crystals.

## Abstract 2

The crystal structure of datiscetin, isolated from the ethyl acetate fraction of *Datisca cannabina* L., and its monohydrate were determined by single-crystal X-ray diffraction analysis.

## 1. Chemical context

*Datisca cannabina* L. (commonly known as false hemp) is a shrub of the family Datiscaceae that resembles hemp (*Cannabis sativa* L.) in many aspects, including its general morphology and the arrangement of its leaves. It is a robust single genus of perennial plant that reaches up to 1–2 m in height and is predominantly found in riparian environments (Holmes & Blizzard, 2010 ▸; Bohmer *et al.*, 2002 ▸). The family Datiscaceae includes three genera and four species, the genus Datisca consists of two species: *D. glomerata* (Presl) Baill., found in California, and *D. cannabina* L., which grows in the area from southwest Asia to Crete (Christopher, 1973 ▸). The aerial parts of the plant are rich in biologically active com­pounds, including flavonoids (17%), tannins (2.9%), cou­marins (0.9–1.5%) and alkaloids (0.31%). *Datisca cannabina* L. is used as a medicinal raw material in the production of Datiscan, a preparation containing a com­plex of flavonoids. This formulation is recommended as part of combination therapy for digestive disorders, including gastric ailments, scrofulous conditions and gastrointestinal diseases accom­panied by smooth muscle spasms. In traditional medicine, infusions and decoctions prepared from the aerial parts of the plant are employed as diuretics, expectorants and laxatives. Moreover, the plant has been reported to exhibit a range of biological activities, such as anti­oxidant, anti-inflammatory, anti­bacterial and anti­carcinogenic properties (Ahmad *et al.*, 2008 ▸). Additionally, the roots are used for obtaining a yellow dye for colouring wool and silk, while the stems provide bast fiber suitable for netting (Muhammed *et al.*, 2012 ▸). In this work, we report the crystal structures of datiscetin (**I**) and its monohydrate (**II**) isolated from *Datisca cannabina* L.
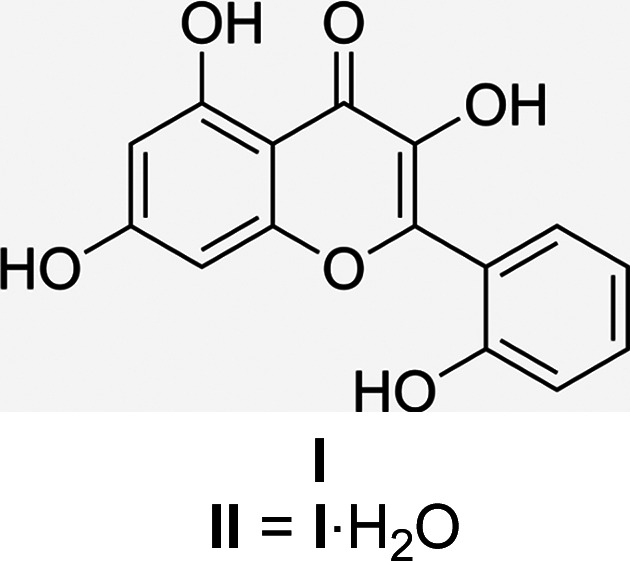


## 2. Structural commentary

**Figure 1:**
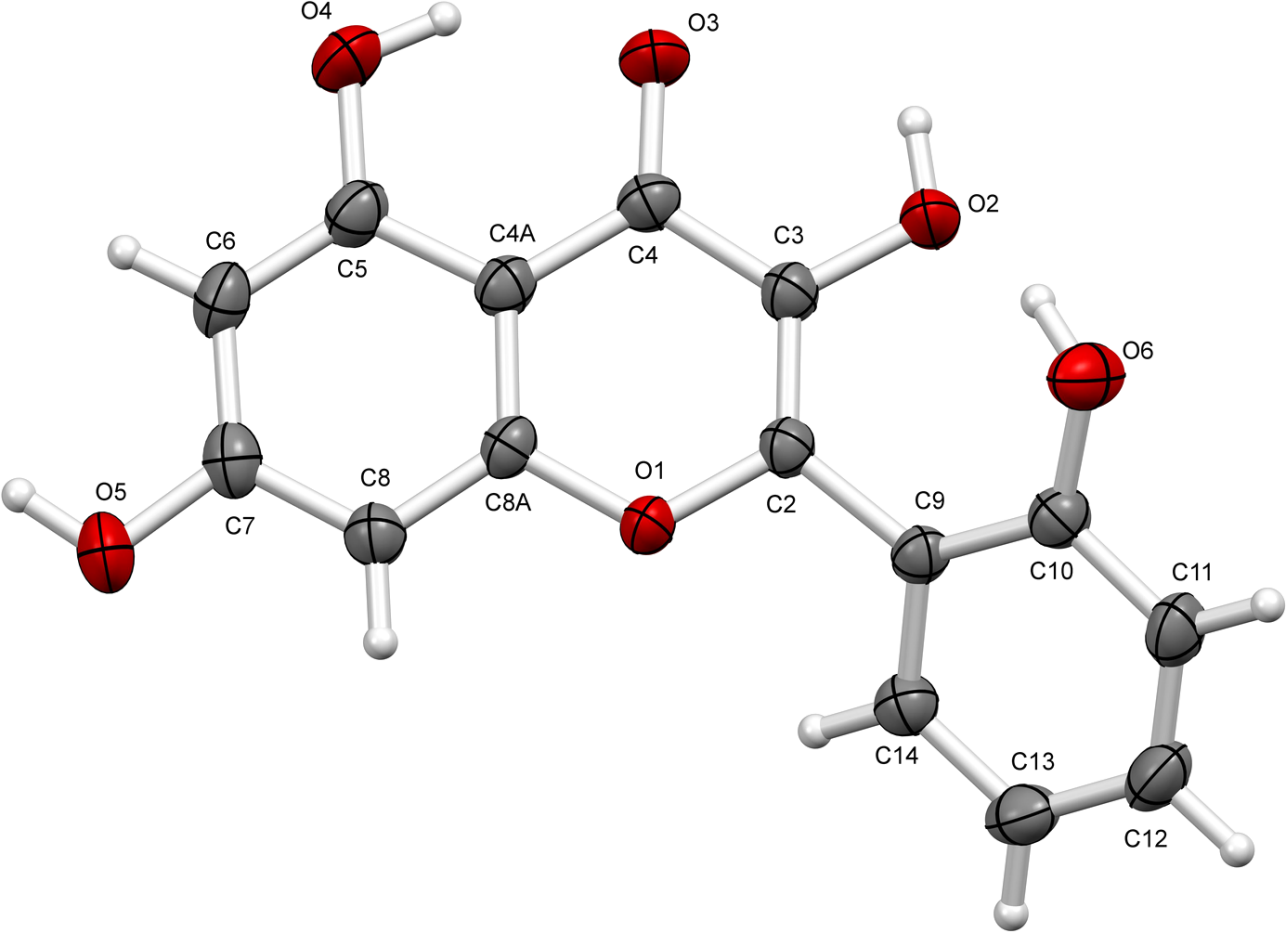
The mol­ecular structure of **I**. Displacement ellipsoids are drawn at the 50% probability level.

Solvent (hydrate)-free crystals (**I**) of datiscetin were obtained from a chloro­form–methanol (7:3 *v*/*v*) mixture. The mol­ecular structure of datiscetin is shown in Fig. 1 ▸. The chromenone ring system and the phenyl ring are planar, with r.m.s. deviations of 0.008 and 0.005 Å, respectively, and the dihedral angle between them is 38.3°. The torsion angle between these two rings is stabilized by an intra­molecular O6—H6⋯O2 hy­dro­gen bond formed between the hydroxyl group of the phenyl ring and the hydroxyl group located at position 3 [2.625 (2) Å and 155 (3)°]. The hydroxyl group at position 5 in the mol­ecule also forms an intra­molecular O4—H4⋯O3 hy­dro­gen bond with the carbonyl group [2.631 (2) Å and 155 (3)°] Table 1 ▸.

**Figure 2:**
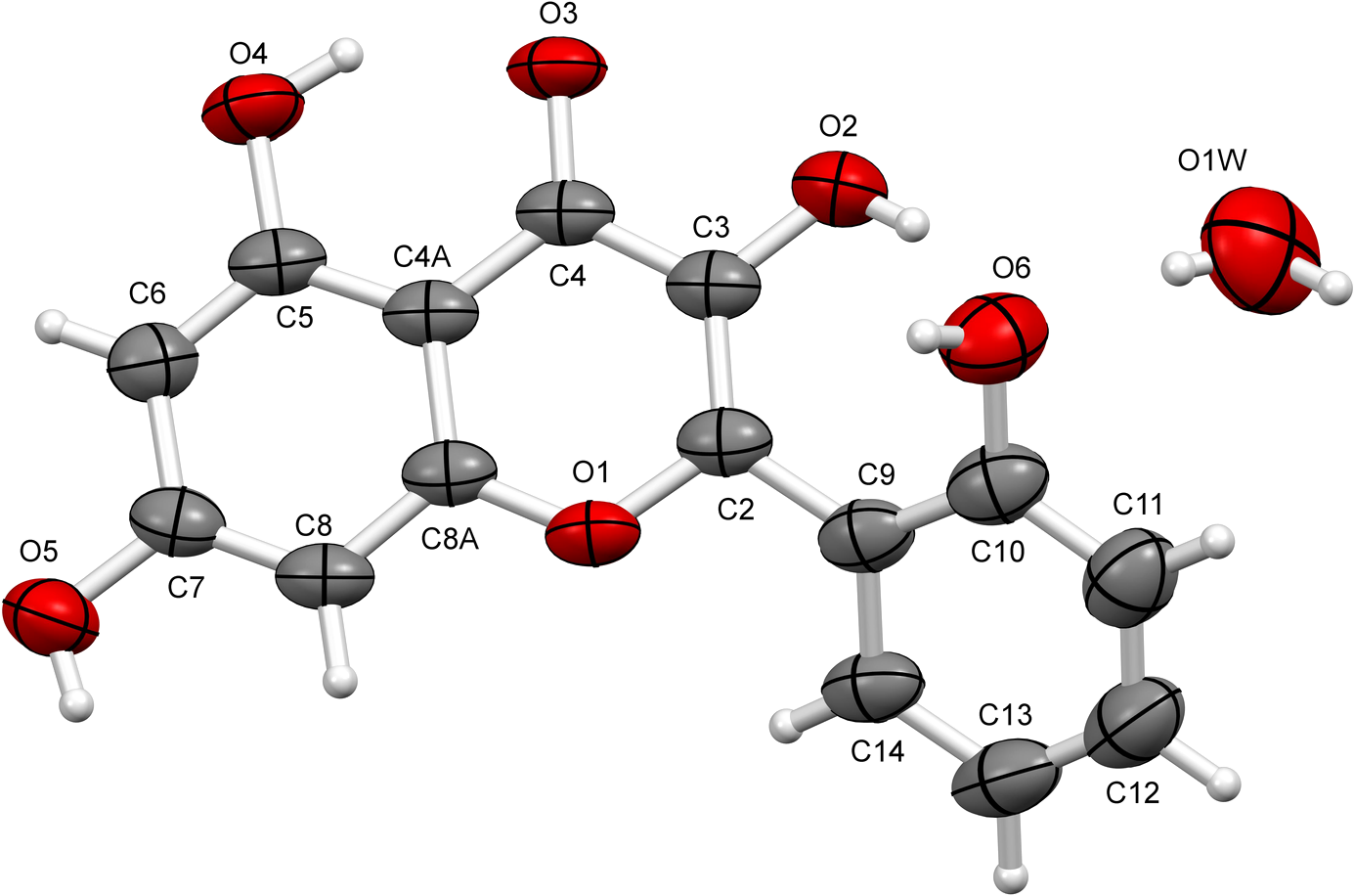
The mol­ecular structure of **II**. Displacement ellipsoids are drawn at the 50% probability level

Datiscetin monohydrate crystals (**II**) were obtained from methanol at room temperature and crystallize in the ortho­rhom­bic space group *Pna*2_1_. The asymmetric unit consists of one flavonoid mol­ecule and one water mol­ecule (Fig. 2 ▸). The chromenone ring system and the phenyl ring are planar, with r.m.s. deviations of 0.015 and 0.004 Å, respectively, and the dihedral angle between them is 38.7°, which is the same as that observed in crystal form **I**, and is stabilized by an intra­molecular O2—H2⋯O6 hy­dro­gen bond formed between the hydroxyl group of the phenyl ring and the hydroxyl group located at position 3 [2.609 (3) Å and 153°; Table 2 ▸].

## 3. Supra­molecular features

**Figure 3:**
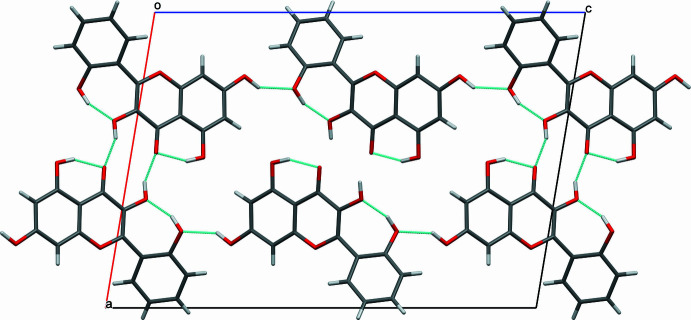
Packing of mol­ecules of **I**, viewed along the *b* axis, showing the formation of a hydrogen-bonded macrocycle consisting of six molecules.

**Figure 4:**
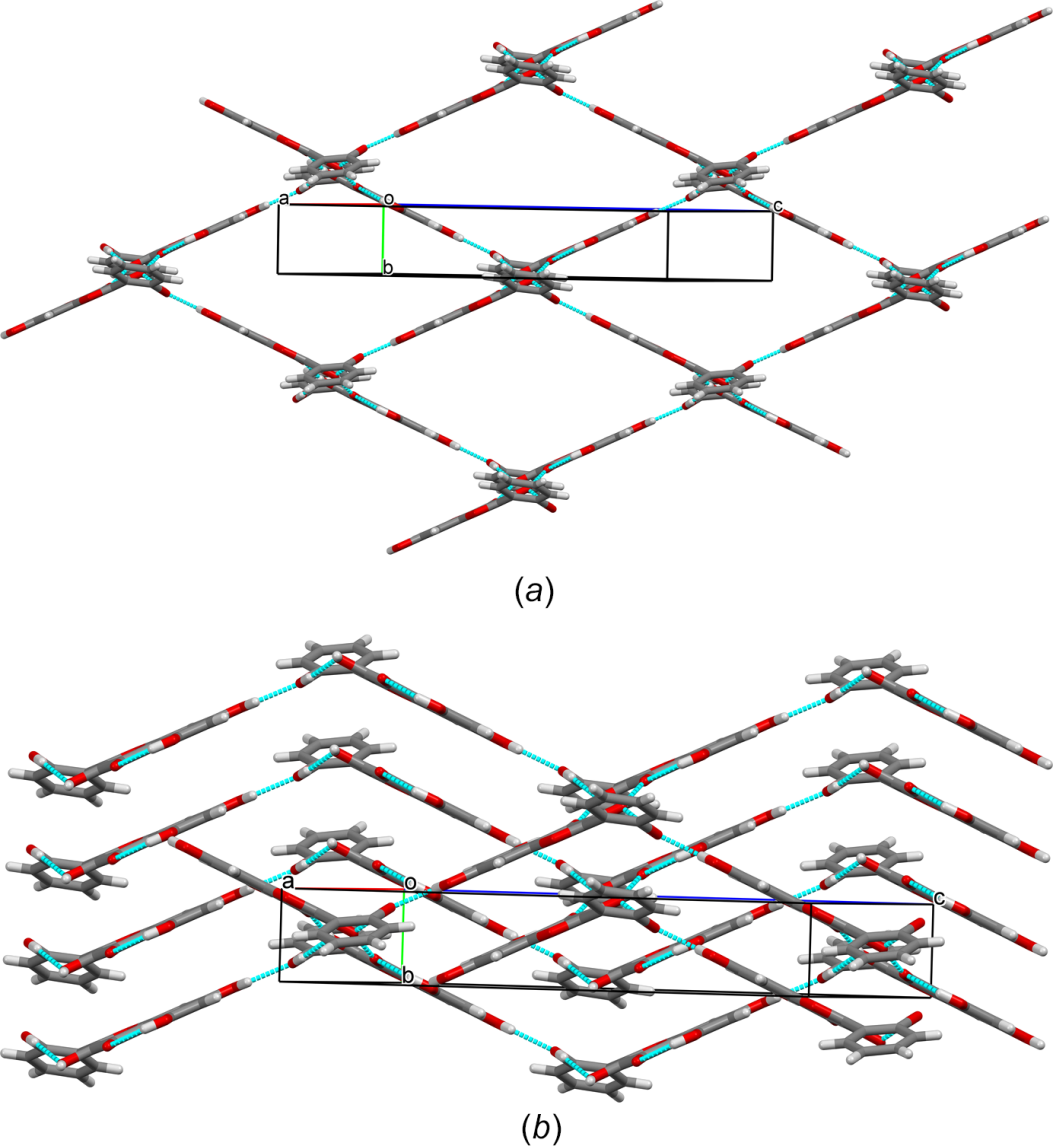
(*a*) Formation of a hy­dro­gen-bonded network, where the hy­dro­gen-bonded rings consist of six mol­ecules of datiscetin. (*b*) Translation (stacking) of the network along the *b* axis. In both cases, the packing is shown along the [40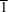] direction.

**Figure 5:**
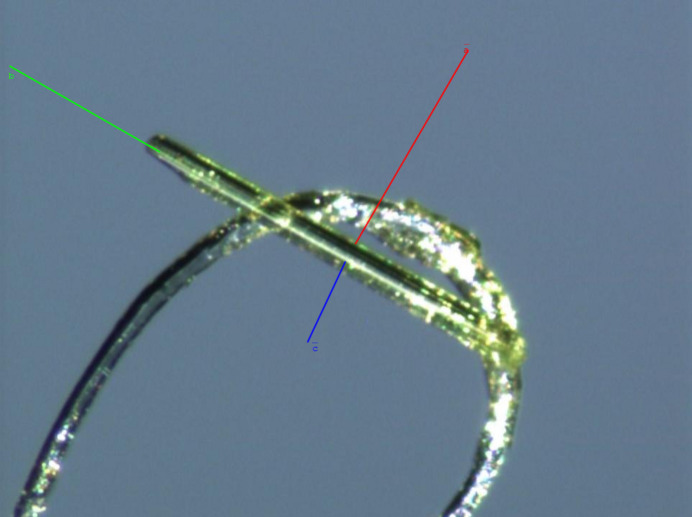
Single crystal of **I** exhibiting a needle-like morphology.

The crystal of **I** possesses an inversion centre, therefore, the crystals contain ‘left’ and ‘right’ mol­ecules with respect to the orientation of the chromenone and phenyl rings. In crystal form **I**, the ‘left’ and ‘right’ mol­ecules related by the inversion centre form hy­dro­gen-bonded dimers through the hydroxyl at position 3 and carbonyl groups [0.85 (3), 1.86 (3), 2.6094 (19) Å and 147 (3)°]. The dimers are connected by hy­dro­gen bonds *via* the hydroxyl groups at position 7 and on the phenyl ring, leading to the formation of a two-dimensional supra­molecular network in the crystal (Table 1 ▸, Fig. 3 ▸). The supra­molecules formed from ‘supercells’ [Fig. 4 ▸(*a*)] are further packed along the *b* axis through π–π stacking inter­actions, with centroid-to-centroid distances equal to the unit-cell translation along the *b* axis [3.7601 (1) Å], with a slippage of 1.59 Å [Fig. 4 ▸(*b*)]. This particular stacking corresponds to the preferential growth direction during crystallization, leading to the formation of needle-shaped single crystals (Fig. 5 ▸).

**Figure 6:**
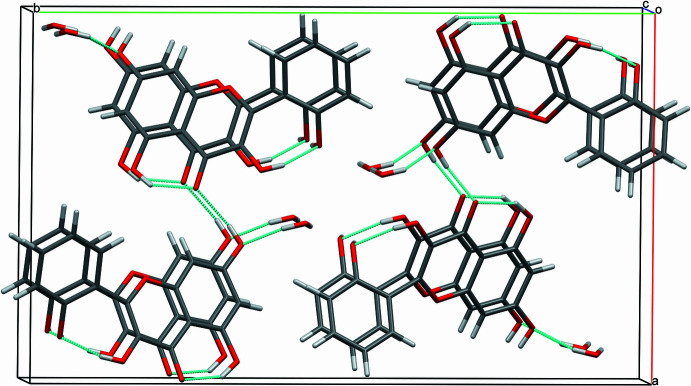
Packing of mol­ecules and hy­dro­gen bonding in **II**.

Packing analysis of the crystal structure of **II** shows that flavonoid mol­ecules related by glide-plane symmetry are con­nected through an O5—H5⋯O3^i^ hy­dro­gen bond [O5⋯O3^i^ = 2.803 (3) Å and 166°; symmetry code: (i) *x* − 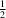, −*y* + 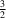, *z* + 1]. Water mol­ecules bridge flavonoid mol­ecules stacked along the *c* axis [O1*W*⋯ O5^ii^ = 3.281 (5) Å and O1*W*⋯ O5^iii^ = 2.934 (4) Å; symmetry codes: (ii) −*x* + 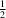, *y* − 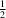, *z* − 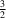; (iii) −*x* + 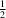, *y* − 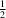, *z* − 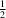] (Table 2 ▸, Fig. 6 ▸). It should be noted that, due to the difficulty of experimentally determining the coordinates of hydrogen atoms in water molecules, the observed other short Ow⋯O distances may indicate the possible presence of alternative hydrogen bonds, not taken into account in the table

**Figure 7:**
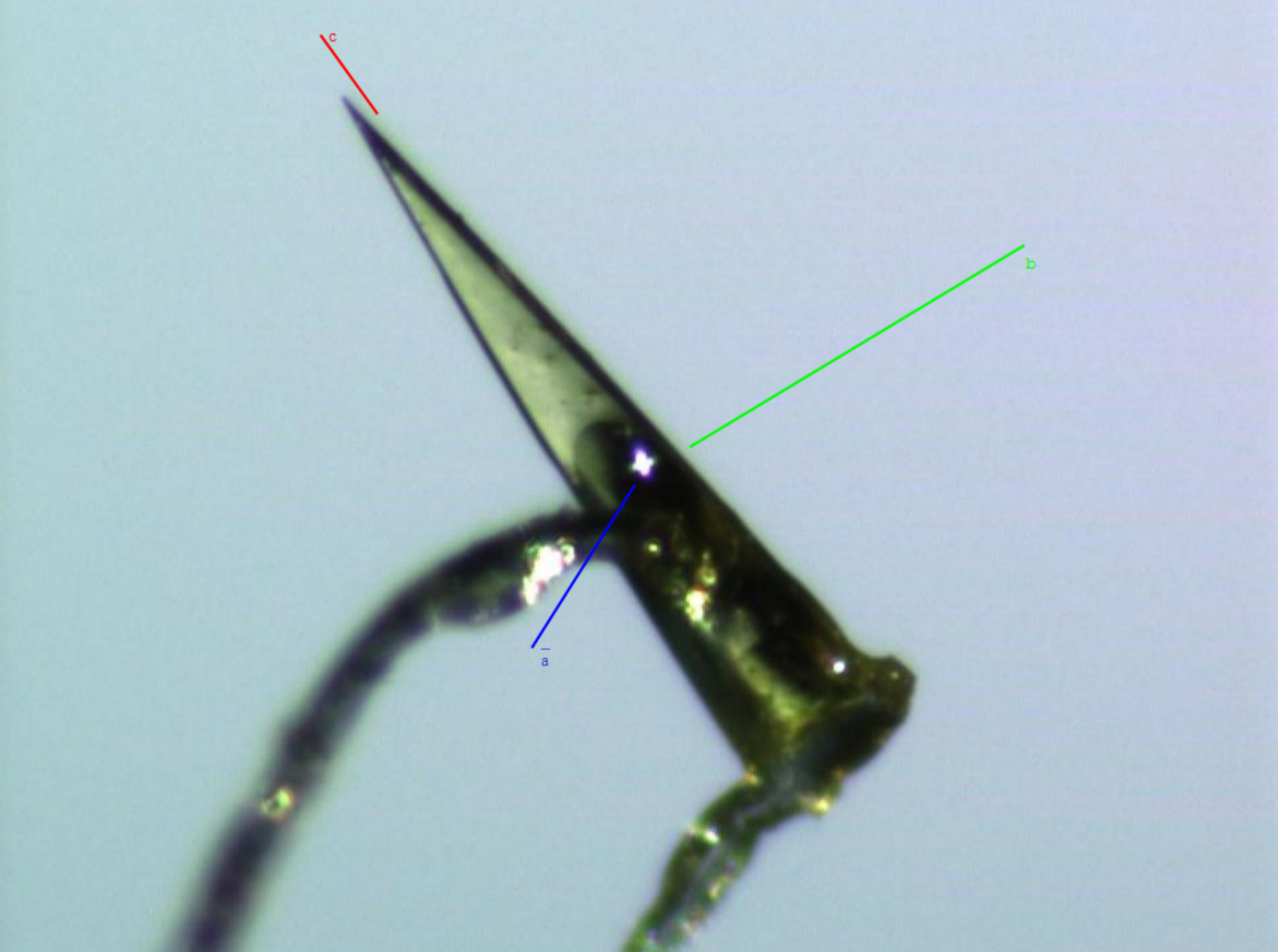
Single crystal of **II** exhibiting a conical-needle-shaped morphology.

The stacking of mol­ecules along the *c* axis corresponds to the preferential growth direction during crystallization, resulting in the formation of conical-needle-shaped single crystals along this axis (Fig. 7 ▸).

## 4. Database survey

A search of the Cambridge Structural Database (CSD, Version 5.41, including the update of January 2020; Groom *et al.*, 2016 ▸) for flavonoids with a 2-phenyl substituent yields more than 1400 hits.

## 5. Isolation and crystallization

### 5.1. Plant material

The above-ground parts (leaves, flowers and stems) of *Datisca cannabina* L. were collected in the Tashkent region, Uzbekistan, in October 2024, during the seed-bearing period. The species identification was confirmed by com­paring the col­lected specimen with herbarium material of *Datisca cannabina* L. preserved at the Central Herbarium of Uzbekistan. The taxonomic identification was carried out by A. M. Nigmatullaev, Senior Researcher at the Laboratory of Biology of Medicinal and Technical Plants, S. Yu. Yunusov Institute of the Chemistry of Plant Substances, Academy of Sciences of the Republic of Uzbekistan.

### 5.2. Extraction and isolation

The freshly collected air-dried powdered plant material (3.0 kg) was extracted ten times by percolation with 75% ethanol. The combined concentrated viscous extract was con­secutively partitioned with solvents of increasing polarity between chloro­form, ethyl acetate and *n*-butanol. The ethyl acetate fraction was adsorbed onto silica gel (1:1 *v*/*v*, 99 g) and subjected to column chromatography (165 × 4.5 cm) with stepwise elution using chloro­form and chloro­form–methanol mixtures (9:1, 8:2 and 7:3 *v*/*v*). The obtained mixture of flavonoids was further separated into individual com­pounds by mol­ecular weight using a Sephadex LH-20 column with methanol as the eluent, yielding 14.3 g of datiscetin.

### 5.3. NMR spectroscopy

NMR spectra were recorded on a JNM-ECZ600R spectrometer (JEOL, Japan) operating at 600 MHz for ^1^H and 150 MHz for ^13^C, using DMSO-*d*_6_ (Cambridge Isotope Laboratories, Inc., USA) as solvent. Tetra­methyl­silane (TMS, 0 ppm) served as the inter­nal standard for ^1^H NMR and ^13^C NMR, the residual solvent signal of DMSO-*d*_6_ (39.52 ppm relative to TMS) was used. Spectral data were processed with *MestReNova* software (Version 14.2.0; Mestrelab Research S.L., Santi­ago de Compostela, Spain).

^1^H NMR (600 MHz, CD_3_OD, ppm δ, J/Hz): 7.55 (1H, *dd*, *J* = 7.8, 1.7, H-6′), 7.37 (1H, *ddd*, *J* = 8.3, 7.3, 1.7, H-4′), 7.00 (1H, *ddd*, *J* = 7.8, 7.3, 1.0, H-5′), 6.98 (1H, *dd*, *J* = 8.3, 1.0, H-3′), 6.34 (1H, *d*, *J* = 2.1, H-8), 6.19 (1H, *d*, *J* = 2.1, H-6).

^13^C NMR (150 MHz, CD_3_OD, ppm. δ): 149.09 (C-2), 137.76 (C-3), 177.98 (C-4), 162.88 (C-5), 99.34 (C-6), 165.74 (C-7), 94.62 (C-8), 159.16 (C-9), 105.22 (C-10), 119.89 (C-1′), 156.37 (C-2′), 118.13 (C-3′), 132.93 (C-4′), 120.90 (C-5′), 131.35 (C-6′).

## 6. Refinement

Crystal data, data collection and structure refinement details are summarized in Table 3 ▸. For **I** and **II**, the H atoms bonded to C atoms were placed in calculated positions and refined to ride on their parent atoms: C—H = 0.93 Å with *U*_iso_(H) = 1.2*U*_eq_(C) for aromatic H atoms. Hydroxyl H atoms in **I** were located using electron-density difference maps, and were refined freely. For **II**, H atoms of the hydroxyl groups and water mol­ecule were placed in calculated positions.

## Supplementary Material

Crystal structure: contains datablock(s) global, h200, h149. DOI: 10.1107/S2056989026004056/nx2034sup1.cif

Structure factors: contains datablock(s) h200. DOI: 10.1107/S2056989026004056/nx2034h200sup2.hkl

Structure factors: contains datablock(s) h149. DOI: 10.1107/S2056989026004056/nx2034h149sup3.hkl

Supporting information file. DOI: 10.1107/S2056989026004056/nx2034h200sup4.cml

Supporting information file. DOI: 10.1107/S2056989026004056/nx2034h149sup5.cml

CCDC references: 2524294, 2524293

Additional supporting information:  crystallographic information; 3D view; checkCIF report

## Figures and Tables

**Table 1:** Hydrogen-bond geometry (Å, °) for **I**[Chem scheme1]

*D*—H⋯*A*	*D*—H	H⋯*A*	*D*⋯*A*	*D*—H⋯*A*
O2—H2⋯O3^i^	0.85 (3)	1.85 (3)	2.6094 (18)	147 (3)
O4—H4⋯O3	0.91 (3)	1.78 (3)	2.631 (2)	155 (3)
O5—H5⋯O6^ii^	0.91 (4)	1.98 (4)	2.7778 (19)	146 (3)
O6—H6⋯O2	0.89 (3)	1.79 (3)	2.6253 (19)	155 (3)

Symmetry codes: (i) 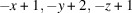; (ii) 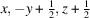.

**Table 2:** Hydrogen-bond geometry (Å, °) for **II**[Chem scheme1]

*D*—H⋯*A*	*D*—H	H⋯*A*	*D*⋯*A*	*D*—H⋯*A*
O2—H2⋯O6	0.82	1.85	2.609 (3)	153
O4—H4⋯O3	0.82	1.91	2.639 (3)	147
O5—H5⋯O3^i^	0.82	2.00	2.803 (3)	166
O1*W*—H1*WB*⋯O5^ii^	0.85	2.76	3.281 (5)	121
O1*W*—H1*WB*⋯O5^iii^	0.85	2.28	2.934 (4)	134

Symmetry codes: (i) 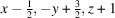; (ii) 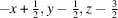; (iii) 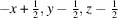.

**Table 3:** Experimental details For all structures: *Z* = 4. Experiments were carried out with Cu *K*α radiation using a Bruker D8 VENTURE dual wavelength Mo/Cu diffractometer. Absorption was corrected for by multi-scan methods, (*SADABS2016*; Krause *et al.*, 2015 ▸). H atoms were treated by a mixture of independent and constrained refinement.

	**I**	**II**
Crystal data		
Chemical formula	C_15_H_10_O_6_	C_15_H_10_O_6_·H_2_O
*M*_r_	286.23	304.25
Crystal system, space group	Monoclinic, *P*2_1_/*c*	Orthorhombic, *Pna*2_1_
Temperature (K)	293	291
*a*, *b*, *c* (Å)	15.0296 (5), 3.7601 (1), 21.6148 (7)	14.0628 (15), 23.714 (3), 3.9274 (5)
α, β, γ (°)	90, 99.453 (2), 90	90, 90, 90
*V* (Å^3^)	1204.93 (6)	1309.7 (3)
μ (mm^−1^)	1.06	1.06
Crystal size (mm)	0.50 × 0.08 × 0.05	0.65 × 0.14 × 0.07

Data collection		
*T*_min_, *T*_max_	0.592, 0.753	0.66, 0.93
No. of measured, independent and observed [*I* > 2σ(*I*)] reflections	26195, 2220, 2026	8711, 2305, 2142
*R*_int_	0.052	0.038
(sin θ/λ)_max_ (Å^−1^)	0.603	0.602

Refinement		
*R*[*F*^2^ > 2σ(*F*^2^)], *wR*(*F*^2^), *S*	0.046, 0.141, 1.17	0.043, 0.116, 0.93
No. of reflections	2220	2305
No. of parameters	199	207
No. of restraints	0	1
Δρ_max_, Δρ_min_ (e Å^−3^)	0.39, −0.37	0.25, −0.21
Absolute structure	–	Flack *x* determined using 811 quotients [(*I*^+^) − (*I*^−^)]/[(*I*^+^) + (*I*^−^)] (Parsons *et al.*, 2013 ▸)
Absolute structure parameter	–	0.25 (10)

Computer programs: *APEX5* (Bruker, 2023 ▸), *SAINT* (Bruker, 2019 ▸), *SHELXT2018* (Sheldrick, 2015*a* ▸), *SHELXL2014* (Sheldrick, 2015*b* ▸), *Mercury* (Macrae *et al.*, 2020 ▸) and *publCIF* (Westrip, 2010 ▸).

## supplementary crystallographic information

### 3,5,7-Trihydroxy-2-(2-hydroxyphenyl)chromen-4-one (h200) Crystal data

**Table:**

C_15_H_10_O_6_	*F*(000) = 592
*M**_r_* = 286.23	*D*_x_ = 1.578 Mg m^−^^3^
Monoclinic, *P*2_1_/*c*	Cu *K*α radiation, λ = 1.54178 Å
*a* = 15.0296 (5) Å	Cell parameters from 8258 reflections
*b* = 3.7601 (1) Å	θ = 4.2–68.2°
*c* = 21.6148 (7) Å	µ = 1.06 mm^−^^1^
β = 99.453 (2)°	*T* = 293 K
*V* = 1204.93 (6) Å^3^	Needle, colourless
*Z* = 4	0.50 × 0.08 × 0.05 mm

### 3,5,7-Trihydroxy-2-(2-hydroxyphenyl)chromen-4-one (h200) Data collection

**Table:**

Bruker D8 VENTURE dual wavelength Mo/Cu diffractometer	2220 independent reflections
Radiation source: microfocus X-ray source, Incoatec IµS 3.0 Microfocus Source	2026 reflections with *I* > 2σ(*I*)
Mirror monochromator	*R*_int_ = 0.052
Detector resolution: 7.3910 pixels mm^-1^	θ_max_ = 68.3°, θ_min_ = 3.0°
ω–φ scans	*h* = −18→17
Absorption correction: multi-scan (SADABS2016; Krause *et al.*, 2015)	*k* = −4→4
*T*_min_ = 0.592, *T*_max_ = 0.753	*l* = −26→26
26195 measured reflections	

### 3,5,7-Trihydroxy-2-(2-hydroxyphenyl)chromen-4-one (h200) Refinement

**Table:**

Refinement on *F*^2^	Hydrogen site location: inferred from neighbouring sites
Least-squares matrix: full	H atoms treated by a mixture of independent and constrained refinement
*R*[*F*^2^ > 2σ(*F*^2^)] = 0.046	*w* = 1/[σ^2^(*F*_o_^2^) + (0.079*P*)^2^ + 0.4321*P*] where *P* = (*F*_o_^2^ + 2*F*_c_^2^)/3
*wR*(*F*^2^) = 0.141	(Δ/σ)_max_ < 0.001
*S* = 1.17	Δρ_max_ = 0.39 e Å^−^^3^
2220 reflections	Δρ_min_ = −0.37 e Å^−^^3^
199 parameters	Extinction correction: SHELXL2014 (Sheldrick, 2015*b*), Fc^*^=kFc[1+0.001xFc^2^λ^3^/sin(2θ)]^-1/4^
0 restraints	Extinction coefficient: 0.0098 (12)

### 3,5,7-Trihydroxy-2-(2-hydroxyphenyl)chromen-4-one (h200) Special details

**Table:**

Geometry. All esds (except the esd in the dihedral angle between two l.s. planes) are estimated using the full covariance matrix. The cell esds are taken into account individually in the estimation of esds in distances, angles and torsion angles; correlations between esds in cell parameters are only used when they are defined by crystal symmetry. An approximate (isotropic) treatment of cell esds is used for estimating esds involving l.s. planes.

### 3,5,7-Trihydroxy-2-(2-hydroxyphenyl)chromen-4-one (h200) Fractional atomic coordinates and isotropic or equivalent isotropic displacement parameters (Å^2^)

**Table:**

	*x*	*y*	*z*	*U*_iso_*/*U*_eq_	
O1	0.20372 (8)	0.6063 (3)	0.53557 (5)	0.0305 (3)	
O2	0.36717 (9)	0.9638 (4)	0.44792 (6)	0.0398 (4)	
H2	0.424 (2)	0.986 (7)	0.4589 (6)	0.060*	
O3	0.47468 (8)	0.7623 (4)	0.55722 (6)	0.0463 (4)	
O4	0.49049 (9)	0.4646 (5)	0.66865 (7)	0.0526 (5)	
H4	0.5088 (7)	0.561 (9)	0.6349 (13)	0.079*	
O5	0.21619 (10)	0.1003 (4)	0.73305 (7)	0.0474 (4)	
H5	0.2541 (14)	0.037 (8)	0.7676 (14)	0.071*	
O6	0.25875 (9)	0.6804 (4)	0.35317 (6)	0.0451 (4)	
H6	0.3070 (16)	0.742 (7)	0.3810 (12)	0.068*	
C2	0.24158 (11)	0.7541 (5)	0.48810 (7)	0.0271 (4)	
C3	0.33153 (11)	0.8063 (5)	0.49504 (8)	0.0298 (4)	
C4	0.39130 (11)	0.7049 (5)	0.55145 (8)	0.0310 (4)	
C4A	0.35007 (12)	0.5439 (5)	0.59939 (8)	0.0299 (4)	
C5	0.40008 (12)	0.4289 (5)	0.65774 (8)	0.0349 (4)	
C6	0.35701 (13)	0.2818 (5)	0.70293 (8)	0.0376 (5)	
H6A	0.3899	0.2079	0.7410	0.045*	
C7	0.26335 (13)	0.2443 (5)	0.69115 (8)	0.0351 (4)	
C8	0.21210 (12)	0.3543 (5)	0.63513 (8)	0.0329 (4)	
H8	0.1497	0.3289	0.6280	0.039*	
C8A	0.25639 (12)	0.5021 (5)	0.59043 (8)	0.0284 (4)	
C9	0.17216 (11)	0.8532 (4)	0.43451 (8)	0.0276 (4)	
C10	0.18276 (12)	0.8150 (5)	0.37161 (8)	0.0317 (4)	
C11	0.11160 (13)	0.9013 (6)	0.32428 (8)	0.0393 (5)	
H11	0.1185	0.8731	0.2826	0.047*	
C12	0.03171 (14)	1.0270 (6)	0.33804 (10)	0.0430 (5)	
H12	−0.0152	1.0817	0.3058	0.052*	
C13	0.02049 (13)	1.0730 (5)	0.40003 (10)	0.0396 (5)	
H13	−0.0334	1.1620	0.4095	0.047*	
C14	0.09014 (12)	0.9853 (5)	0.44718 (9)	0.0334 (4)	
H14	0.0824	1.0148	0.4887	0.040*	

### 3,5,7-Trihydroxy-2-(2-hydroxyphenyl)chromen-4-one (h200) Atomic displacement parameters (Å^2^)

**Table:**

	*U*^11^	*U*^22^	*U*^33^	*U*^12^	*U*^13^	*U*^23^
O1	0.0263 (6)	0.0411 (7)	0.0231 (6)	−0.0033 (5)	0.0007 (5)	0.0043 (5)
O2	0.0265 (7)	0.0625 (9)	0.0292 (7)	−0.0102 (6)	0.0007 (5)	0.0095 (6)
O3	0.0252 (7)	0.0706 (10)	0.0408 (8)	−0.0103 (6)	−0.0010 (5)	0.0121 (7)
O4	0.0321 (8)	0.0806 (12)	0.0405 (8)	−0.0066 (7)	−0.0078 (6)	0.0149 (8)
O5	0.0500 (8)	0.0630 (10)	0.0304 (7)	−0.0006 (7)	0.0099 (6)	0.0147 (7)
O6	0.0338 (7)	0.0715 (10)	0.0293 (7)	0.0027 (7)	0.0029 (5)	−0.0133 (7)
C2	0.0277 (8)	0.0308 (8)	0.0227 (8)	−0.0026 (7)	0.0034 (6)	−0.0005 (6)
C3	0.0287 (9)	0.0361 (9)	0.0241 (8)	−0.0040 (7)	0.0028 (7)	0.0001 (7)
C4	0.0262 (8)	0.0358 (9)	0.0296 (9)	−0.0039 (7)	0.0007 (7)	−0.0010 (7)
C4A	0.0293 (9)	0.0330 (9)	0.0259 (8)	−0.0020 (7)	0.0002 (7)	−0.0011 (7)
C5	0.0327 (9)	0.0394 (10)	0.0296 (9)	−0.0018 (7)	−0.0037 (7)	−0.0003 (8)
C6	0.0425 (11)	0.0416 (10)	0.0258 (9)	0.0011 (8)	−0.0031 (7)	0.0045 (8)
C7	0.0445 (11)	0.0343 (9)	0.0269 (9)	−0.0004 (8)	0.0073 (7)	0.0010 (7)
C8	0.0322 (9)	0.0375 (10)	0.0288 (9)	−0.0028 (7)	0.0045 (7)	0.0020 (7)
C8A	0.0297 (9)	0.0309 (9)	0.0228 (8)	0.0000 (7)	−0.0010 (6)	−0.0011 (7)
C9	0.0258 (8)	0.0298 (8)	0.0258 (8)	−0.0045 (6)	0.0003 (6)	0.0012 (7)
C10	0.0296 (9)	0.0364 (10)	0.0282 (9)	−0.0036 (7)	0.0022 (7)	−0.0022 (7)
C11	0.0409 (10)	0.0493 (11)	0.0250 (9)	−0.0048 (9)	−0.0026 (7)	0.0019 (8)
C12	0.0354 (10)	0.0483 (12)	0.0402 (11)	−0.0013 (8)	−0.0092 (8)	0.0105 (9)
C13	0.0280 (9)	0.0418 (11)	0.0475 (11)	0.0032 (8)	0.0020 (8)	0.0040 (9)
C14	0.0298 (9)	0.0388 (10)	0.0313 (9)	−0.0019 (7)	0.0042 (7)	0.0004 (7)

### 3,5,7-Trihydroxy-2-(2-hydroxyphenyl)chromen-4-one (h200) Geometric parameters (Å, º)

**Table:**

O1—C2	1.370 (2)	C5—C6	1.374 (3)
O1—C8A	1.370 (2)	C6—C7	1.396 (3)
O2—C3	1.361 (2)	C6—H6A	0.9300
O2—H2	0.85 (3)	C7—C8	1.387 (3)
O3—C4	1.257 (2)	C8—C8A	1.377 (2)
O4—C5	1.347 (2)	C8—H8	0.9300
O4—H4	0.90 (3)	C9—C14	1.397 (3)
O5—C7	1.352 (2)	C9—C10	1.402 (2)
O5—H5	0.89 (3)	C10—C11	1.392 (3)
O6—C10	1.367 (2)	C11—C12	1.368 (3)
O6—H6	0.89 (3)	C11—H11	0.9300
C2—C3	1.350 (2)	C12—C13	1.389 (3)
C2—C9	1.474 (2)	C12—H12	0.9300
C3—C4	1.442 (2)	C13—C14	1.376 (3)
C4—C4A	1.427 (2)	C13—H13	0.9300
C4A—C8A	1.398 (2)	C14—H14	0.9300
C4A—C5	1.425 (2)		
			
C2—O1—C8A	120.81 (13)	C8—C7—C6	121.86 (17)
C3—O2—H2	109.5	C8A—C8—C7	118.02 (17)
C5—O4—H4	109.5	C8A—C8—H8	121.0
C7—O5—H5	109.5	C7—C8—H8	121.0
C10—O6—H6	109.5	O1—C8A—C8	116.45 (15)
C3—C2—O1	120.48 (15)	O1—C8A—C4A	120.87 (15)
C3—C2—C9	128.12 (15)	C8—C8A—C4A	122.67 (16)
O1—C2—C9	111.33 (14)	C14—C9—C10	118.09 (16)
C2—C3—O2	119.34 (15)	C14—C9—C2	117.99 (15)
C2—C3—C4	121.93 (16)	C10—C9—C2	123.90 (16)
O2—C3—C4	118.72 (15)	O6—C10—C11	116.75 (16)
O3—C4—C4A	123.05 (16)	O6—C10—C9	123.65 (16)
O3—C4—C3	120.72 (16)	C11—C10—C9	119.54 (17)
C4A—C4—C3	116.22 (15)	C12—C11—C10	121.12 (18)
C8A—C4A—C5	117.47 (16)	C12—C11—H11	119.4
C8A—C4A—C4	119.65 (16)	C10—C11—H11	119.4
C5—C4A—C4	122.87 (16)	C11—C12—C13	120.20 (17)
O4—C5—C6	119.70 (17)	C11—C12—H12	119.9
O4—C5—C4A	119.65 (17)	C13—C12—H12	119.9
C6—C5—C4A	120.65 (17)	C14—C13—C12	119.12 (18)
C5—C6—C7	119.34 (16)	C14—C13—H13	120.4
C5—C6—H6A	120.3	C12—C13—H13	120.4
C7—C6—H6A	120.3	C13—C14—C9	121.90 (17)
O5—C7—C8	115.30 (17)	C13—C14—H14	119.0
O5—C7—C6	122.84 (17)	C9—C14—H14	119.0
			
C8A—O1—C2—C3	−1.3 (3)	C2—O1—C8A—C8	−179.56 (15)
C8A—O1—C2—C9	−178.60 (14)	C2—O1—C8A—C4A	0.0 (2)
O1—C2—C3—O2	−177.97 (15)	C7—C8—C8A—O1	179.72 (16)
C9—C2—C3—O2	−1.2 (3)	C7—C8—C8A—C4A	0.2 (3)
O1—C2—C3—C4	1.1 (3)	C5—C4A—C8A—O1	−179.98 (15)
C9—C2—C3—C4	177.86 (17)	C4—C4A—C8A—O1	1.5 (3)
C2—C3—C4—O3	−178.72 (18)	C5—C4A—C8A—C8	−0.5 (3)
O2—C3—C4—O3	0.4 (3)	C4—C4A—C8A—C8	−178.93 (17)
C2—C3—C4—C4A	0.4 (3)	C3—C2—C9—C14	−140.56 (19)
O2—C3—C4—C4A	179.49 (16)	O1—C2—C9—C14	36.4 (2)
O3—C4—C4A—C8A	177.41 (18)	C3—C2—C9—C10	41.0 (3)
C3—C4—C4A—C8A	−1.7 (3)	O1—C2—C9—C10	−141.99 (17)
O3—C4—C4A—C5	−1.0 (3)	C14—C9—C10—O6	−178.23 (17)
C3—C4—C4A—C5	179.91 (16)	C2—C9—C10—O6	0.2 (3)
C8A—C4A—C5—O4	−179.79 (17)	C14—C9—C10—C11	−1.4 (3)
C4—C4A—C5—O4	−1.4 (3)	C2—C9—C10—C11	177.08 (17)
C8A—C4A—C5—C6	0.2 (3)	O6—C10—C11—C12	177.87 (18)
C4—C4A—C5—C6	178.67 (18)	C9—C10—C11—C12	0.8 (3)
O4—C5—C6—C7	−179.73 (18)	C10—C11—C12—C13	0.4 (3)
C4A—C5—C6—C7	0.2 (3)	C11—C12—C13—C14	−1.0 (3)
C5—C6—C7—O5	179.59 (18)	C12—C13—C14—C9	0.4 (3)
C5—C6—C7—C8	−0.5 (3)	C10—C9—C14—C13	0.8 (3)
O5—C7—C8—C8A	−179.78 (16)	C2—C9—C14—C13	−177.76 (17)
C6—C7—C8—C8A	0.3 (3)		

### 3,5,7-Trihydroxy-2-(2-hydroxyphenyl)chromen-4-one (h200) Hydrogen-bond geometry (Å, º)

**Table:**

*D*—H···*A*	*D*—H	H···*A*	*D*···*A*	*D*—H···*A*
O2—H2···O3^i^	0.85 (3)	1.85 (3)	2.6094 (18)	147 (3)
O4—H4···O3	0.91 (3)	1.78 (3)	2.631 (2)	155 (3)
O5—H5···O6^ii^	0.91 (4)	1.98 (4)	2.7778 (19)	146 (3)
O6—H6···O2	0.89 (3)	1.79 (3)	2.6253 (19)	155 (3)

Symmetry codes: (i) −*x*+1, −*y*+2, −*z*+1; (ii) *x*, −*y*+1/2, *z*+1/2.

### 3,5,7-Trihydroxy-2-(2-hydroxyphenyl)chromen-4-one monohydrate (h149) Crystal data

**Table:**

C_15_H_10_O_6_·H_2_O	*D*_x_ = 1.543 Mg m^−^^3^
*M**_r_* = 304.25	Cu *K*α radiation, λ = 1.54178 Å
Orthorhombic, *P**n**a*2_1_	Cell parameters from 4404 reflections
*a* = 14.0628 (15) Å	θ = 7.3–68.1°
*b* = 23.714 (3) Å	µ = 1.06 mm^−^^1^
*c* = 3.9274 (5) Å	*T* = 291 K
*V* = 1309.7 (3) Å^3^	Needle, colourless
*Z* = 4	0.65 × 0.14 × 0.07 mm
*F*(000) = 632	

### 3,5,7-Trihydroxy-2-(2-hydroxyphenyl)chromen-4-one monohydrate (h149) Data collection

**Table:**

Bruker D8 VENTURE dual wavelength Mo/Cu diffractometer	2305 independent reflections
Radiation source: microfocus X-ray source, Incoatec IµS 3.0 Microfocus Source	2142 reflections with *I* > 2σ(*I*)
Mirror monochromator	*R*_int_ = 0.038
Detector resolution: 7.3910 pixels mm^-1^	θ_max_ = 68.3°, θ_min_ = 7.3°
ω–φ scans	*h* = −16→16
Absorption correction: multi-scan (SADABS2016; Krause *et al.*, 2015)	*k* = −25→28
*T*_min_ = 0.66, *T*_max_ = 0.93	*l* = −4→4
8711 measured reflections	

### 3,5,7-Trihydroxy-2-(2-hydroxyphenyl)chromen-4-one monohydrate (h149) Refinement

**Table:**

Refinement on *F*^2^	Hydrogen site location: mixed
Least-squares matrix: full	H atoms treated by a mixture of independent and constrained refinement
*R*[*F*^2^ > 2σ(*F*^2^)] = 0.043	*w* = 1/[σ^2^(*F*_o_^2^) + (0.0954*P*)^2^ + 0.0942*P*] where *P* = (*F*_o_^2^ + 2*F*_c_^2^)/3
*wR*(*F*^2^) = 0.116	(Δ/σ)_max_ = 0.002
*S* = 0.93	Δρ_max_ = 0.25 e Å^−^^3^
2305 reflections	Δρ_min_ = −0.20 e Å^−^^3^
207 parameters	Absolute structure: Flack *x* determined using 811 quotients [(I+)-(I-)]/[(I+)+(I-)] (Parsons *et al.*, 2013)
1 restraint	Absolute structure parameter: 0.25 (10)

### 3,5,7-Trihydroxy-2-(2-hydroxyphenyl)chromen-4-one monohydrate (h149) Special details

**Table:**

Geometry. All esds (except the esd in the dihedral angle between two l.s. planes) are estimated using the full covariance matrix. The cell esds are taken into account individually in the estimation of esds in distances, angles and torsion angles; correlations between esds in cell parameters are only used when they are defined by crystal symmetry. An approximate (isotropic) treatment of cell esds is used for estimating esds involving l.s. planes.

### 3,5,7-Trihydroxy-2-(2-hydroxyphenyl)chromen-4-one monohydrate (h149) Fractional atomic coordinates and isotropic or equivalent isotropic displacement parameters (Å^2^)

**Table:**

	*x*	*y*	*z*	*U*_iso_*/*U*_eq_	
O1	0.21017 (12)	0.67355 (8)	0.4950 (5)	0.0496 (5)	
O2	0.42512 (15)	0.62983 (10)	0.0550 (7)	0.0659 (6)	
H2	0.4119	0.5962	0.0606	0.099*	
O3	0.47772 (13)	0.73003 (8)	0.2957 (6)	0.0594 (5)	
O4	0.43416 (14)	0.82483 (10)	0.6100 (7)	0.0670 (6)	
H4	0.472 (3)	0.7990 (17)	0.501 (15)	0.101*	
O5	0.12647 (14)	0.83978 (9)	1.0763 (7)	0.0663 (6)	
H5	0.0782	0.8212	1.1113	0.099*	
O6	0.36173 (17)	0.53262 (11)	0.2674 (9)	0.0776 (7)	
H6	0.3647	0.5392	0.4721	0.116*	
C2	0.27162 (18)	0.64084 (11)	0.3106 (7)	0.0474 (6)	
C3	0.36183 (17)	0.65904 (12)	0.2473 (7)	0.0492 (6)	
C4	0.39466 (18)	0.71322 (12)	0.3617 (7)	0.0488 (6)	
C4A	0.32752 (17)	0.74680 (12)	0.5469 (7)	0.0465 (6)	
C5	0.34735 (17)	0.80153 (11)	0.6692 (7)	0.0501 (6)	
C6	0.2810 (2)	0.83166 (11)	0.8462 (8)	0.0542 (7)	
H6A	0.2953	0.8675	0.9271	0.065*	
C7	0.19053 (19)	0.80783 (12)	0.9051 (7)	0.0511 (6)	
C8	0.16834 (17)	0.75469 (12)	0.7910 (7)	0.0501 (6)	
H8	0.1090	0.7390	0.8345	0.060*	
C8A	0.23623 (16)	0.72515 (10)	0.6101 (7)	0.0454 (6)	
C9	0.22479 (19)	0.58975 (12)	0.1862 (7)	0.0527 (7)	
C10	0.2694 (2)	0.53740 (13)	0.1606 (8)	0.0611 (8)	
C11	0.2213 (3)	0.49107 (15)	0.0292 (10)	0.0738 (10)	
H11	0.2522	0.4565	0.0125	0.089*	
C12	0.1298 (3)	0.49590 (18)	−0.0747 (11)	0.0772 (10)	
H12	0.0983	0.4646	−0.1618	0.093*	
C13	0.0833 (2)	0.54674 (18)	−0.0524 (9)	0.0729 (9)	
H13	0.0207	0.5498	−0.1264	0.087*	
C14	0.1295 (2)	0.59331 (14)	0.0801 (8)	0.0586 (7)	
H14	0.0972	0.6274	0.0993	0.070*	
O1W	0.4385 (3)	0.43331 (13)	0.1462 (13)	0.1119 (12)	
H1WA	0.4279	0.4453	0.3467	0.168*	
H1WB	0.4247	0.3984	0.1542	0.168*	

### 3,5,7-Trihydroxy-2-(2-hydroxyphenyl)chromen-4-one monohydrate (h149) Atomic displacement parameters (Å^2^)

**Table:**

	*U*^11^	*U*^22^	*U*^33^	*U*^12^	*U*^13^	*U*^23^
O1	0.0353 (9)	0.0610 (10)	0.0525 (11)	−0.0084 (7)	0.0025 (8)	0.0096 (9)
O2	0.0449 (11)	0.0735 (12)	0.0795 (15)	−0.0039 (9)	0.0108 (10)	−0.0067 (12)
O3	0.0327 (9)	0.0747 (11)	0.0708 (13)	−0.0106 (8)	0.0067 (8)	0.0018 (10)
O4	0.0434 (10)	0.0699 (12)	0.0878 (17)	−0.0176 (8)	0.0086 (11)	0.0024 (12)
O5	0.0467 (11)	0.0675 (11)	0.0846 (16)	0.0038 (9)	0.0102 (11)	0.0011 (12)
O6	0.0639 (13)	0.0716 (13)	0.0973 (19)	−0.0017 (10)	−0.0159 (14)	0.0092 (15)
C2	0.0370 (12)	0.0619 (14)	0.0435 (13)	−0.0044 (10)	−0.0025 (10)	0.0124 (12)
C3	0.0367 (12)	0.0630 (14)	0.0480 (14)	−0.0027 (10)	−0.0001 (11)	0.0094 (13)
C4	0.0323 (12)	0.0667 (15)	0.0475 (14)	−0.0048 (11)	−0.0005 (10)	0.0129 (13)
C4A	0.0334 (12)	0.0607 (14)	0.0455 (13)	−0.0044 (10)	−0.0014 (10)	0.0134 (12)
C5	0.0341 (11)	0.0607 (14)	0.0555 (15)	−0.0064 (10)	−0.0040 (11)	0.0125 (13)
C6	0.0470 (15)	0.0543 (14)	0.0612 (18)	−0.0028 (11)	−0.0028 (13)	0.0071 (13)
C7	0.0369 (13)	0.0630 (15)	0.0532 (14)	0.0042 (11)	−0.0008 (11)	0.0108 (13)
C8	0.0326 (12)	0.0636 (15)	0.0540 (15)	−0.0034 (10)	−0.0006 (11)	0.0133 (13)
C8A	0.0340 (12)	0.0580 (13)	0.0442 (13)	−0.0046 (9)	−0.0035 (11)	0.0141 (12)
C9	0.0458 (14)	0.0670 (16)	0.0454 (14)	−0.0118 (11)	0.0028 (11)	0.0090 (12)
C10	0.0553 (17)	0.0714 (17)	0.0565 (17)	−0.0116 (13)	−0.0028 (14)	0.0107 (15)
C11	0.081 (2)	0.0707 (18)	0.070 (2)	−0.0196 (16)	0.0053 (18)	−0.0015 (17)
C12	0.074 (2)	0.091 (2)	0.0660 (19)	−0.032 (2)	0.0016 (18)	−0.0084 (18)
C13	0.0527 (17)	0.107 (3)	0.0587 (18)	−0.0280 (19)	−0.0013 (14)	0.0002 (18)
C14	0.0426 (14)	0.0837 (19)	0.0497 (15)	−0.0141 (13)	0.0021 (12)	0.0066 (15)
O1W	0.119 (3)	0.0767 (16)	0.140 (3)	0.0237 (16)	0.004 (2)	0.015 (2)

### 3,5,7-Trihydroxy-2-(2-hydroxyphenyl)chromen-4-one monohydrate (h149) Geometric parameters (Å, º)

**Table:**

O1—C8A	1.355 (3)	C6—C7	1.411 (4)
O1—C2	1.369 (3)	C6—H6A	0.9300
O2—C3	1.357 (4)	C7—C8	1.373 (4)
O2—H2	0.8200	C8—C8A	1.381 (4)
O3—C4	1.261 (3)	C8—H8	0.9300
O4—C5	1.360 (3)	C9—C10	1.395 (4)
O4—H4	0.92 (6)	C9—C14	1.405 (4)
O5—C7	1.356 (4)	C10—C11	1.390 (5)
O5—H5	0.8200	C11—C12	1.355 (6)
O6—C10	1.369 (4)	C11—H11	0.9300
O6—H6	0.8200	C12—C13	1.375 (6)
C2—C3	1.363 (3)	C12—H12	0.9300
C2—C9	1.463 (4)	C13—C14	1.383 (5)
C3—C4	1.437 (4)	C13—H13	0.9300
C4—C4A	1.433 (4)	C14—H14	0.9300
C4A—C8A	1.405 (3)	O1W—H1WA	0.8500
C4A—C5	1.412 (4)	O1W—H1WB	0.8499
C5—C6	1.365 (4)		
			
C8A—O1—C2	121.18 (19)	C7—C8—C8A	118.4 (2)
C3—O2—H2	109.5	C7—C8—H8	120.8
C5—O4—H4	109.5	C8A—C8—H8	120.8
C7—O5—H5	109.5	O1—C8A—C8	116.3 (2)
C10—O6—H6	109.5	O1—C8A—C4A	121.2 (2)
O1—C2—C3	120.3 (2)	C8—C8A—C4A	122.5 (3)
O1—C2—C9	111.2 (2)	C10—C9—C14	117.5 (3)
C3—C2—C9	128.4 (3)	C10—C9—C2	124.0 (2)
O2—C3—C2	123.4 (3)	C14—C9—C2	118.6 (3)
O2—C3—C4	114.8 (2)	O6—C10—C11	120.7 (3)
C2—C3—C4	121.7 (3)	O6—C10—C9	118.6 (3)
O3—C4—C4A	122.6 (3)	C11—C10—C9	120.7 (3)
O3—C4—C3	121.0 (3)	C12—C11—C10	120.5 (4)
C4A—C4—C3	116.3 (2)	C12—C11—H11	119.8
C8A—C4A—C5	117.2 (2)	C10—C11—H11	119.8
C8A—C4A—C4	119.2 (3)	C13—C12—C11	120.5 (3)
C5—C4A—C4	123.6 (2)	C13—C12—H12	119.8
O4—C5—C6	119.2 (3)	C11—C12—H12	119.8
O4—C5—C4A	119.5 (3)	C12—C13—C14	120.0 (3)
C6—C5—C4A	121.3 (2)	C12—C13—H13	120.0
C5—C6—C7	119.3 (3)	C14—C13—H13	120.0
C5—C6—H6A	120.3	C13—C14—C9	120.8 (3)
C7—C6—H6A	120.3	C13—C14—H14	119.6
O5—C7—C8	121.6 (2)	C9—C14—H14	119.6
O5—C7—C6	117.2 (3)	H1WA—O1W—H1WB	104.5
C8—C7—C6	121.2 (3)		
			
C8A—O1—C2—C3	1.9 (3)	C2—O1—C8A—C8	179.5 (2)
C8A—O1—C2—C9	−174.3 (2)	C2—O1—C8A—C4A	−0.5 (3)
O1—C2—C3—O2	−177.3 (2)	C7—C8—C8A—O1	−178.3 (2)
C9—C2—C3—O2	−1.7 (5)	C7—C8—C8A—C4A	1.7 (4)
O1—C2—C3—C4	−1.8 (4)	C5—C4A—C8A—O1	178.4 (2)
C9—C2—C3—C4	173.8 (2)	C4—C4A—C8A—O1	−1.0 (4)
O2—C3—C4—O3	−2.7 (4)	C5—C4A—C8A—C8	−1.6 (4)
C2—C3—C4—O3	−178.6 (3)	C4—C4A—C8A—C8	179.0 (2)
O2—C3—C4—C4A	176.1 (2)	O1—C2—C9—C10	−143.9 (3)
C2—C3—C4—C4A	0.3 (4)	C3—C2—C9—C10	40.2 (4)
O3—C4—C4A—C8A	179.9 (3)	O1—C2—C9—C14	37.2 (3)
C3—C4—C4A—C8A	1.1 (4)	C3—C2—C9—C14	−138.7 (3)
O3—C4—C4A—C5	0.6 (4)	C14—C9—C10—O6	−179.0 (3)
C3—C4—C4A—C5	−178.3 (2)	C2—C9—C10—O6	2.1 (5)
C8A—C4A—C5—O4	−178.9 (3)	C14—C9—C10—C11	1.1 (4)
C4—C4A—C5—O4	0.4 (4)	C2—C9—C10—C11	−177.8 (3)
C8A—C4A—C5—C6	1.0 (4)	O6—C10—C11—C12	179.7 (4)
C4—C4A—C5—C6	−179.6 (3)	C9—C10—C11—C12	−0.4 (5)
O4—C5—C6—C7	179.4 (3)	C10—C11—C12—C13	0.1 (6)
C4A—C5—C6—C7	−0.6 (4)	C11—C12—C13—C14	−0.7 (6)
C5—C6—C7—O5	−178.8 (3)	C12—C13—C14—C9	1.4 (5)
C5—C6—C7—C8	0.7 (4)	C10—C9—C14—C13	−1.6 (4)
O5—C7—C8—C8A	178.2 (3)	C2—C9—C14—C13	177.3 (3)
C6—C7—C8—C8A	−1.3 (4)		

### 3,5,7-Trihydroxy-2-(2-hydroxyphenyl)chromen-4-one monohydrate (h149) Hydrogen-bond geometry (Å, º)

**Table:**

*D*—H···*A*	*D*—H	H···*A*	*D*···*A*	*D*—H···*A*
O2—H2···O6	0.82	1.85	2.609 (3)	153
O4—H4···O3	0.82	1.91	2.639 (3)	147
O5—H5···O3^i^	0.82	2.00	2.803 (3)	166
O1*W*—H1*WB*···O5^ii^	0.85	2.76	3.281 (5)	121
O1*W*—H1*WB*···O5^iii^	0.85	2.28	2.934 (4)	134

Symmetry codes: (i) *x*−1/2, −*y*+3/2, *z*+1; (ii) −*x*+1/2, *y*−1/2, *z*−3/2; (iii) −*x*+1/2, *y*−1/2, *z*−1/2.
